# Retraction: MiR-206 reduced the malignancy of hepatocellular carcinoma cells *in vitro* by inhibiting MET and CTNNB1 gene expressions

**DOI:** 10.1039/d1ra90022f

**Published:** 2021-01-21

**Authors:** Laura Fisher

**Affiliations:** Royal Society of Chemistry Thomas Graham House, Science Park, Milton Road Cambridge CB4 0WF UK advances-rsc@rsc.org

## Abstract

Retraction of ‘MiR-206 reduced the malignancy of hepatocellular carcinoma cells *in vitro* by inhibiting MET and CTNNB1 gene expressions’ by Qiang He *et al.*, *RSC Adv.*, 2019, **9**, 1717–1725, DOI: 10.1039/C8RA09229J

The Royal Society of Chemistry hereby wholly retracts this *RSC Advances* article due to concerns with the reliability of the data. The images in the article were screened by an image integrity expert who raised concerns with the integrity of the western blot panels. Many of the published western blot panels share the same background, indicating that the images have been manipulated.

In Fig 2F, 5 of the panels share the same background.

In Fig 3D, all 4 panels share the same background.

In Fig 5D, 3 of the panels share the same background.

In Fig 6A and B, all 4 panels share the same background.

The authors were asked to provide the raw data for this article, but did not respond. Given the significance of the concerns about the validity of the data, and the lack of raw data, the findings presented in this paper are not reliable.

The authors have been informed but have not responded to any correspondence regarding the retraction.

Signed: Laura Fisher, Executive Editor, *RSC Advances*

Date: 7^th^ January 2021

## Supplementary Material

